# Comparative Biomechanical Strategies of Running Gait Among Healthy and Recently Injured Pediatric and Adult Runners

**DOI:** 10.3390/bioengineering12090937

**Published:** 2025-08-30

**Authors:** Cole Verble, Ryan M. Nixon, Lydia Pezzullo, Matthew Martenson, Kevin R. Vincent, Heather K. Vincent

**Affiliations:** 1 Exercise and Functional Fitness Laboratory, Department of Physical Medicine and Rehabilitation, University of Florida, Gainesville, FL 32610, USA; cverble@ufl.edu (C.V.); lpezzullo@ufl.edu (L.P.); matthewmartenson@medicine.ufl.edu (M.M.); heatherketelaar@gmail.com (H.K.V.); 2 The Orthopaedic Institute (TOI), Alachua, FL 32615, USA; krvincent1@gmail.com

**Keywords:** running, injury, adolescent, pediatric, gait, loading rate, kinematics

## Abstract

Biomechanical strategies of running gait were compared among healthy and recently injured pediatric and adult runners (N = 207). Spatiotemporal, kinematic, and kinetic parameters (ground reaction force [GRF], vertical average loading rate [VALR]) and leg stiffness (K_vert_) were obtained during running on an instrumented treadmill with simultaneous 3D-motion capture. Significant age X injury interactions existed for cadence, peak GRF, and peak joint angles in stance. Cadence was fastest in healthy adults and 2–3% lower in other groups (*p* = 0.049). Injured adults exhibited higher variance in stance and swing time, whereas injured pediatric runners had lower variance in these measures (*p* < 0.05). Peak GRF was highest in non-injured adults (2.6–2.7 BW) and lowest in injured adults (2.4 BW; *p* < 0.05). VALRs (BW/s) were higher among pediatric groups, irrespective of injury (*p* < 0.05). The interaction for ankle dorsiflexion/plantarflexion moment was significant (*p* = 0.05). Healthy pediatric runners produced more plantarflexion than all other groups (*p* = 0.026). Pelvis rotation was highest in healthy pediatric runners and lowest in healthy adults (17.3° versus 12.0°; *p* = 0.036). Pediatric runners did not leverage force-dampening strategies, but reduced gait cycle time variance and controlled pelvic rotation. Injured adults had lower GRF and longer stance time, indicating a shift toward force mitigation during stance. Age-specific rehabilitation and gait retraining approaches may be warranted.

## 1. Introduction

Over the last decade, participation in running among the pediatric population has steadily increased by an estimated 10%. During the 2023–2024 academic year, 423,350 U.S. high school students participated in cross-country, and 1.13 million participated in track and field [1]. According to the 2022 USA Global Runner Survey, adults aged 25–54 years comprise 48% of the overall running population [2]. Industry survey data indicate growing participation in running among the older adult demographic, with a 59% global growth in running club participation among adults [3]. Despite the popularity of running, there are several inherent risks of musculoskeletal injury, including biomechanical patterns and musculoskeletal loading. Several studies have determined the proportion of injuries with incidence rates ranging from 3.2% to as high as 84.9% depending on the study sample [4,5,6]. To ensure that participation rates remain high among pediatric and adult runners, it is imperative to understand the age differences in biomechanical strategies when runners are injured and when they are healthy. Pediatric patients are not ‘small adults’ and are likely to have different running responses after an injury. A strong understanding of age-related mechanics is essential for clinicians and therapists to guide the return-to-run process after injury, enabling them to: (1) inform age-specific clinical approaches to gait retraining by tailoring loading rate reduction strategies and movement cueing techniques for younger versus older athletes, and (2) refine the goals and content of rehabilitation programs in distance runners with different injuries by age.

Characterization studies reveal differences in running biomechanical parameters between pediatric and adult runners, which may be due to the ongoing development of motor control and coordination [7]. Compared to adults and adolescents in later maturational stages, younger adolescents undergoing developmental changes exhibit shorter stride lengths and higher cadence [8]. In addition, pre-pubertal runners demonstrate higher variability with specific metrics such as hip flexion, hip adduction, hip internal rotation, and knee flexion than post-pubertal runners [9]. As young runners mature, some investigators have documented a reduction in cadence [10] and an increase in peak vertical ground reaction forces (vGRF) [11] which are related to lower extremity overuse running injuries [12]. Pediatric runners control forward momentum during gait termination by adjusting step frequency rather than joint moment adaptations, a strategy less commonly seen in adults [13]. Spatiotemporal gait parameters also differ significantly between children and adults. Differences such as variable stride length and width, as well as labile velocities, can alter a runner’s center of mass [13]. Cesar et al. [13] found that pediatric runners utilized less stable running mechanics to build momentum during the initial start of a run. Adolescents exhibit asymmetry in their running gait at different speeds, which may contribute to an increased risk of musculoskeletal imbalances over time [14].

Adult runners exhibit refined and stable gait mechanics, characterized by greater efficiency in energy transfer [15]. This stability has been attributed to well-developed neuromuscular coordination and more consistent activation of lower limb musculature [7]. In our recent age comparison of natural running biomechanical asymmetries, we found that runners <18 years produced greater, more variable sagittal hip and knee excursions, greater knee adduction/abduction variability, and ankle joint plantarflexion moment variability than adults [16]. Maturation-related changes in knee and hip strength can also influence running biomechanics; specifically, younger runners may have less effective, dynamically strong hip abductors and knee extensors, potentially predisposing them to altered gait mechanics and increasing their susceptibility to injury [17].

While previous works have characterized running biomechanics in different age populations with different injuries, there is no direct comparison of these features according to age and injury status. As such, clinicians often extrapolate data from injured adults to develop interventions and cues for gait retraining in children and adolescents. To determine whether biomechanical strategies of running motion differ between injured and healthy non-injured pediatric and adult runners, a comprehensive analysis of temporospatial, kinetic, and kinematic features is needed. Therefore, the purpose of this investigation was to compare running gait biomechanical parameters between healthy and recently injured pediatric and adult runners. We hypothesized that pediatric injured runners (<18 yrs) would produce higher GRFs, load rates, and greater lower extremity joint excursions during a typical gait cycle compared to injured adults (25–35 yrs).

## 2. Materials and Methods

### 2.1. Study Design

**Figure 1:**
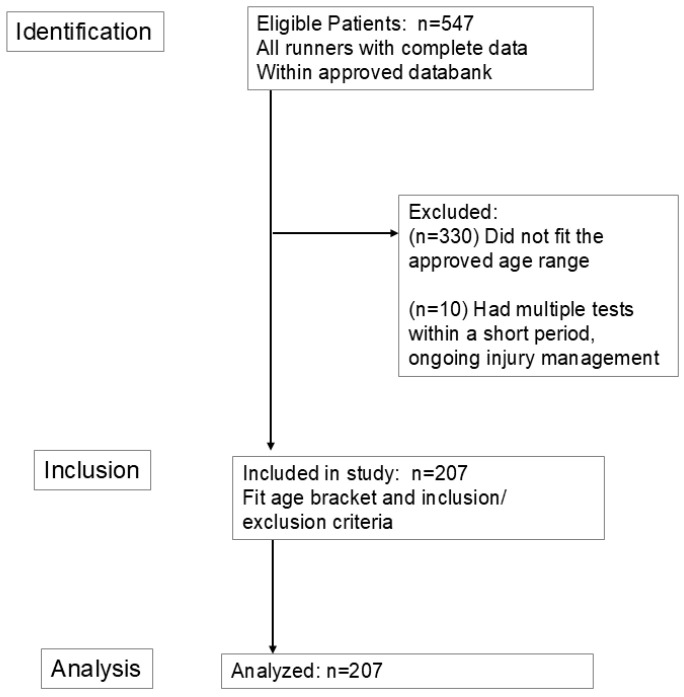
STROBE study flow diagram for observational studies.

This was a cross-sectional study of the running gait in pediatric and adult runners without a running-related injury. This study and its procedures followed the guidelines for the Declaration of Helsinki’s Protection of Human Subjects. The study was approved by the University of Florida Institutional Review Board (study #202500580). The manuscript follows the recommended format for observational research described by the statement in Strengthening the Reporting of Observational Studies in Epidemiology (STROBE) [18]. The Study Flow Diagram is presented in Figure 1.

### 2.2. Setting

The Exercise Medicine and Functional Fitness Laboratory is located within a quaternary health care facility and provides gait services among several performance-based and injury prevention options. After physician referral or self-referral into the laboratory, the team offers 3D running motion analysis, functional movement assessment screen to identify kinetic chain deficits, training program recommendations, gait retraining with cueing, counseling on return-to-run programs, shoe wear selection, and therapeutic exercise to runners of all ages and experience levels (amateur to professional athletes).

### 2.3. Participants

Participants were a convenience sample obtained from our departmental research databank (Study #202101632) from January 2014 to 5 April 2025. Inclusion criteria were: (1) aged <18 years or 25–35 years; (2) injury free for over 6 months (“non-injured”) or injured within the preceding six months (“recently injured”); and (3) previous experience of more than 60 min of using treadmills as part of training, which is considered ample for treadmill accommodation [19]. Exclusion criteria were: (1) aged 19–24.9 years or older than 35 years; (2) major traumatic musculoskeletal injury (i.e., anterior cruciate ligament rupture); and (3) other pre-existing neurological or orthopedic conditions that interfered with normal gait (i.e., scoliosis, sciatic pain, ataxias, cerebral palsy) or a previous history of orthopedic trauma with resultant persistent motion aberrations. Runners were categorized into four groups by age group (<18 years or 25–35 years) and injury status (not injured, recently injured). Exclusion of the ages 19–24 was to ensure that we assessed runners with stable mechanics who were in distinct age groups and not transitioning from adolescence to adulthood, and to avoid the systemic age changes in running form beyond the age of 35 years. A total of 207 runners were included in this analysis.

### 2.4. Participant Characteristics

Characteristics were collected from a comprehensive intake form based on our published recommendations for runner assessment [20]. Sections included characteristics and medical history, presence and type of injury if present, training history (volume, type, cross-training activities, strengthening exercises), shoe wear characteristics (weight, heel-to-toe drop, heel height), and orthotics if applicable. Injury types present in this analysis included recent history of lower extremity stress fractures (healed and cleared to run), tendinopathies (Achilles, flexor hallucis longus, iliotibial band, extensor hallucis longus), plantar fasciitis, and hip labral pain or tear. The current training status for competition was obtained (yes, no). Each runner used their regular, preferred training shoes for the testing to minimize any acute effects of changing footwear on biomechanical responses.

### 2.5. Data Sources and Measurements

Data were acquired from a comprehensive health history intake and biomechanical running gait analyses.

**Figure 2:**
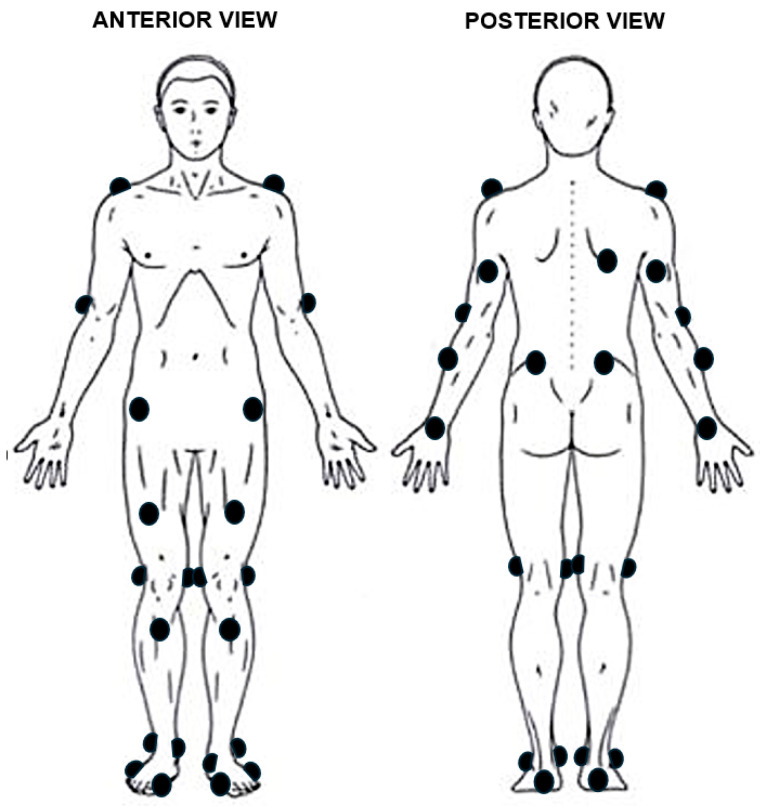
Anatomical location of the skin-based retroreflective markers. Black circle = marker.

Initial Procedures and Instrumentation for Running Analysis. Motion during running at self-selected speed was captured using a high-speed, seven-camera 3D optical motion analysis system (Motion Analysis Corp., Santa Rosa, CA, USA). Motion data sampled at 200 Hz were synchronized with force plate data collected from an instrumented treadmill (AMTI, Watertown, MA, USA) at 1200 Hz [21,22]. The method of Kadaba et al. [23] was used to apply 33 retroreflective markers on anatomical landmarks, as shown in Figure 2 [24]. Markers were placed onto acromion processes, triceps (midway between elbow and shoulder), lateral elbow condyles, radial forearms midway between elbow and wrist), dorsal wrists, posterior superior iliac spine, anterior superior iliac spine, anterior bilaterally on the thigh, medial and lateral condyles of the femur, tibial tuberosity, medial and lateral malleoli, calcaneus, lateral to the head of the fifth metatarsal, and medial to the base of the hallux. One offset marker was placed on the right scapular inferior angle. Prior to data collection, a static calibration trial was collected to develop the computer model of each runner in a neutral anatomical position using commercial software (Cortex version 9.2, Motion Analysis Corp., Santa Rosa, CA, USA).

The optical motion cameras captured the static positions of the retroreflective markers. Each marker was identified by the software in the baseline skeletal model using the known marker set. Body segment lengths and joint centers were established based on the anatomical marker placement by the Cortex software version 9.2.

Data Collection during Running and Post-Processing. Runners ran at a self-selected velocity, defined as the typical pace used for long-distance running. After acclimating to the treadmill for eight minutes to ensure dynamic stabilization of kinematics, slow-motion videos were captured for reference in the sagittal and frontal planes (240 Hz). Between minutes 9 and 10, a 10-s sample of data was captured, with an average of 12–14 strides.

Joint angles at initial foot contact for the ankle, knee, hip, and pelvis were determined from the software tracking of the retroreflective markers in space in each frame for each trial. Using the foot markers, the ankle angle at foot strike was calculated using the foot segment and the ground at initial contact relative to the natural angle during standing [25]. The reference point for joint angles was established at 90° as vertical for the knee and hip, and 0° as horizontal for the ankle angle. The pelvis segment was generated from a digitization of the anterior and posterior superior iliac spine markers, and the pelvis anterior inclination was expressed relative to the horizontal as 0° of anterior tilt.

Several standard spatiotemporal, spatial, and kinematic variables were determined. Bone models were developed for each runner to establish the individual center of mass (COM) location using commercially available software (Visual3D, version.17.1 C-Motion, Inc., Germantown, MD, USA). [21,26] Marker data were filtered at 9 Hz with a fourth-order, low-pass Butterworth filter. Studies in gait and running analysis recommend cutoff frequencies in the 6–12 Hz range based on residual analysis and signal-to-noise optimization. A 9 Hz cutoff is a well-balanced choice, effectively capturing running biomechanics while minimizing soft tissue artifacts. Bone models were generated using the methods of de Leva et al. [24]. Gait cycle time is presented in percent (0% = initial foot contact, 100% = same foot contact post-swing phase). Cadence (steps/min) and the vertical displacement of the COM (the difference between the minimal and maximal vertical height of the COM during a gait cycle) were calculated. The distance between two successive placements of the same foot was defined as the stride length. The medial-lateral distance between the proximal end position of the foot at the foot strike and the end position of the foot at the next contralateral foot strike was calculated as the stride width. Stance time was defined as the duration that each foot remained in contact with the treadmill. The medial-lateral range of motion (ROM) excursion of the COM was calculated as the shift in the medial-lateral positions of the estimated COM during an average gait cycle [27].

The foot strike type was determined by the angle between the foot segment and the horizontal ground at foot contact and was visually confirmed with high-speed videos. Runners were categorized into rearfoot and non-rearfoot strikers. Joint excursions of the ankle, knee, and hip represented the angular excursion of the joint in the sagittal, frontal, and transverse planes during an average gait cycle for the ankle, knee, hip, and pelvis. The pelvis was developed from the anterior and posterior superior iliac spine markers, and the anterior inclination was expressed relative to the horizontal as 0° of anterior tilt.

Force data were collected from the instrumented treadmill at a frequency of 1200 Hz. A threshold of 20 N GRF was used to set the initial foot contact and toe-off. GRF data were low-pass filtered with a cutoff frequency of 40 Hz using a 4th-order Butterworth filter. Processed, filtered treadmill data were integrated by Cortex software with the motion data, and three-dimensional kinetics were determined via full inverse dynamics calculations in Visual 3D. The peak GRF, the vertical average loading rate (VALR), and vertical impulses were normalized to body weight. The vertical component was chosen because it is the most widely studied aspect of loading in comparative literature. VALR was calculated using the slope of the ΔF/Δt of the most linear portion of the force curve, where ΔF is the change in vertical force and Δt is the change in time (between 20–80% of the first rise to the peak of the vertical GRF [28]) or vertical GRF at 13% of stance in case the initial peak was absent.

Vertical stiffness was estimated using the following: K_vert_ = F_max_/Δy, where F_max_ is the peak vertical force and Δy is the maximum displacement of the COM. Leg vertical stiffness, or K_vert_, describes the interaction of the load placed on the leg and the resulting limb and central nervous system’s response to attenuate that load. The maximum joint moments in the sagittal plane (flexion/extension) and frontal planes (inversion/eversion, adduction/abduction) were determined for the ankle, knee, and hip. Joint moments were normalized to body mass multiplied by leg length. The preprocessed filtered treadmill data were combined with the motion data. Three-dimensional kinetics were determined via full inverse dynamics calculations implemented in Visual 3D.

### 2.6. Statistical Analyses

Statistical analyses were performed using SPSS version 29.0 (IBM, Armonk, New York, NY, USA). Normality of the data (skewness and kurtosis) was assessed using Kolmogorov-Smirnov tests. Descriptive statistics were calculated on all study variables and demographics. The assumptions of normality were tested on demographic, anthropometric, and training history continuous variables to determine whether differences existed between age brackets. Chi-square tests (χ^2^) were used to determine if there were differences in categorical variables among the four age groups. Univariate analyses of covariance (ANCOVA) were applied to test group differences for biomechanical variables, where the between-group factors were age (<18 years, 19–35 years) and injury status (non-injured, injured). Based on published evidence that running velocity and sex can affect the biomechanics of running [29], these variables were entered as covariates. The eta squared (η^2^) values were provided to show the effect sizes for continuous variables; values of 0.01, 0.06, and 0.14 represented negligible to small, medium, and large effects [25]. Phi values (Φ) were determined as effect sizes for categorical variables. Statistical significance was established in advance at *p* < 0.05.

## 3. Results

### 3.1. Characteristics

Participant characteristics are provided in Table 1. The healthy adult group consisted of more females than the other groups (both *p* < 0.05). A lower proportion of injured adults were currently competing compared to the remaining groups (*p* < 0.001). Compared to adults, a lower proportion of both pediatric groups participated in yoga/Pilates and strength training, and overall, the pediatric group wore shoes with a larger heel-to-toe drop (all *p* < 0.05). The non-injured pediatric group had the highest proportion of rearfoot strikers (*p* = 0.042). The injured pediatric group was characterized by a higher proportion of bone injuries and a lower proportion of soft tissue injuries compared to the adult injured group (*p* < 0.05). The injured adult group participated less in competition around the time of injury (*p* = 0.023).

### 3.2. Temporal Spatial Parameters

Table 2 provides values for key temporal and spatial parameters for the four study groups. Of all these parameters, cadence was found to have a significant age group X injury status interaction term; the adult non-injured runners had the highest cadence, whereas the injured adult comparators had the lowest cadence (*p* = 0.044). Age group X injury status interactions were detected for both stance time and swing time variability (both *p* < 0.05); the injured pediatric group demonstrated less variability with injury, whereas the adult runners demonstrated more variability with injury compared to their non-injured comparative groups. A main effect of age was found for stance and swing times, where pediatric runners demonstrated shorter stance times and longer swing times than adults (both *p* < 0.05). Pediatric runners also had narrower stride width than adults, independent of injury (*p* = 0.042). The effect sizes for the presented variables were negligible to small (η^2^ range = 0.001–0.019).

### 3.3. Kinetic Parameters and Leg Stiffness

Key kinetic variables and K_vert_ are shown in Table 3. Significant interaction terms between age group X injury status were found for left and right peak vertical GRF values, where non-injured adults produced the highest values among all groups (*p* < 0.05). While the interaction term was not significant for VALR, there was a significant main effect for age, where pediatric runners produced higher VALR than adults, irrespective of injury status (both *p* < 0.05). Similarly, a main effect of age was found for K_vert_, where pediatric runners had lower stiffness values irrespective of injury status compared to adults (*p* = 0.017). The effect sizes for these variables were negligible-small to large (η^2^ range = 0.001–0.120).

**Figure 3:**
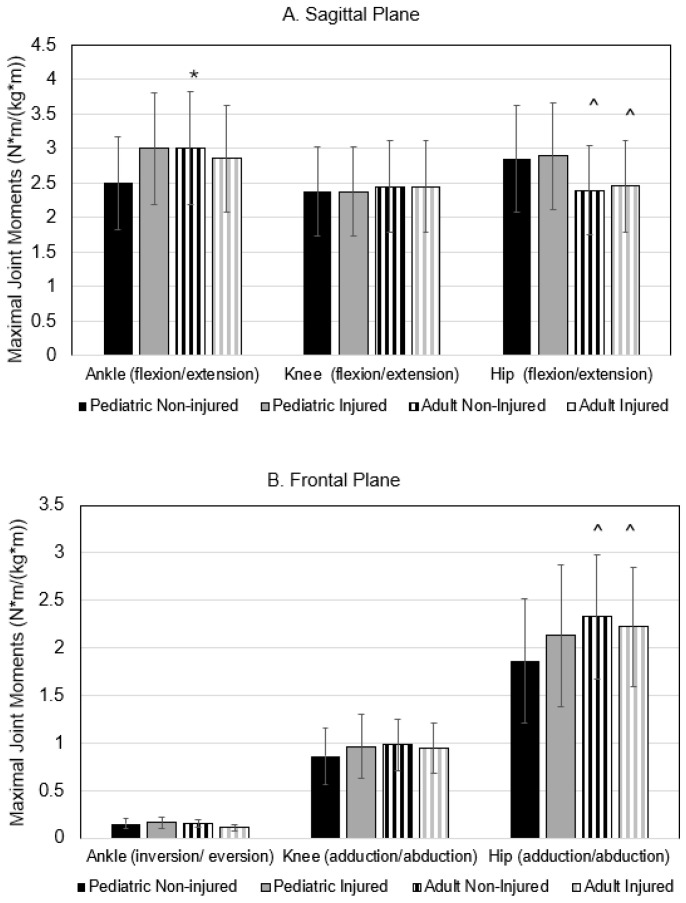
Maximal joint moments in the sagittal plane (Panel **A**) and the frontal plane (Panel **B**). Values are means SD. * significant interaction at *p* < 0.05; ^ = significant main effect of age group at *p* < 0.05.

The maximal joint moments are presented in Figure 3. The group X injury interaction term was significant for ankle joint flexion moment, where the value was highest in the non-injured adult group, but lowest in the non-injured pediatric group (*p* = 0.050; η^2^ = 0.023). A main effect for age was also found for maximal hip flexion/extension and adduction/abduction moments; irrespective of injury status, adults produced lower flexion/extension and greater adduction/abduction moments compared to pediatric runners (both *p* < 0.05).

### 3.4. Kinematic Parameters

Joint excursions during a typical gait cycle are shown for all three planes of motion in Table 4. A significant age group X injury status interaction term was found for sagittal ankle motion, where the largest excursion was produced by non-injured pediatric runners and the smallest was produced by the non-injured adults (*p* = 0.026). Similarly, there was a significant interaction term for pelvis rotation excursion, with excursions being the greatest in the non-injured pediatric group and least in the non-injured adults (*p* = 0.016). Effect sizes were both considered small (η^2^ = 0.031 and 0.036, respectively).

## 4. Discussion

This analysis revealed biomechanical strategies of running motion between injured and healthy non-injured pediatric and adult runners. The novel findings were that injured adults may use a different biomechanical strategy than pediatric runners to run without injury and after a recent injury. Overall, the pediatric runners in the present study are characterized by higher VALR, greater hip flexion/extension and hip adduction/abduction moments, greater pelvis rotation, narrow stride width, and lower K_vert_ values compared to adults. In the presence of injury, maximal vertical GRF values were lower. At the same time, pelvic rotation was greater after injury in adults. Injured pediatric runners adopted a higher cadence but had lower movement excursion at the pelvis and lower variance in stance and swing time than their non-injured counterparts. Collectively, these data suggest that different biomechanical targets for rehabilitative gait retraining may apply to pediatric runners and adults.

### 4.1. Age-Related Running Differences

Currently, differences between healthy adult runners and healthy pediatric runners are quantified by limited data. Overall, our pediatric runners exhibited greater variability in specific metrics of running gait, including stance and swing time. Close et al. [9] reported that prepubertal runners display kinematic inconsistency compared to their post-pubertal counterparts in hip flexion, hip internal rotation, and knee flexion. This finding may be due to the ongoing development of motor control and coordination. Younger runners undergo biomechanical adaptations as they mature [12]. A 10-year-old will have different running patterns versus a 14-year-old due to lengthening of long bones and improved neuromuscular coordination as a child progresses through puberty. With musculoskeletal maturity, adults exhibit more refined and stable gait mechanics, characterized by greater efficiency in energy transfer and reduced kinematic variability [30]. Stability of gait patterns is primarily attributed to well-developed neuromuscular coordination and consistent activation of lower limb musculature [5]. Proximal hip and knee strength deficits among younger runners increase the likelihood of poorer running mechanics [7]. High hip and knee extension muscle strength among young adults is related to faster cadence, longer stride length, and lower peak vGRF compared to low leg strength [17]. In our study, we found that healthy pediatric runners had the highest vGRF, which may be due to underdeveloped lower extremity dynamic eccentric strength and neuromotor coordination. While we did not measure muscle strength directly, our pediatric runners reported less participation in programmatic strength training activity for lower and upper body than adults (Table 1). Thus, emphasis should be on strengthening exercise and neuromotor activity in the core and lower extremities to improve kinematic stability.

### 4.2. Injury-Related Running Differences

Running experience may influence self-awareness of running form and may alter how runners respond to injury [9]. In our study, adults had an average of five additional years of experience and ran at slower velocities. Through experience, adult runners may have learned adaptive responses to offset loading stress on painful or recently injured tissues. In our injured adults, VALR was 13% higher, while K_vert_ was approximately 9% lower than in healthy adults, with a corresponding 4% increase in knee flexion during stance (Table 3); this response could signify the enhanced dependence on knee flexion to modulate impact with the ground. Our pediatric runners increased K_vert_. The relationships of load rates and leg stiffness with injury onset are inconsistent, with some evidence supporting associations [31] whereas others do not [32,33]. However, in response to injury, leg muscle stiffness may change to help reduce discomfort or prevent re-injury [34]. Shear wave elastography measures have captured lower K_vert_ among adults with medial tibial stress syndrome (with or without pain) compared to healthy controls [35]. Although adult runners are reported to learn to intuitively adopt compensatory running patterns when injured, it is clinically important to observe whether this adaptation is beneficial or if it might worsen the injury or cause a new one. Blyton et al. found that the compensatory running gait persisted after the injury had healed in some athlete populations [36]. Clinicians and therapists need to understand maladaptive gait patterns. Identifying which age groups could benefit from interventions to correct maladaptive running gait before it becomes habitual may provide valuable information for the running community.

Injury type may also influence adaptive biomechanics. Evidence shows that VALR is elevated in some specific soft tissue injuries (plantar fasciitis, patellofemoral pain) but is lower in bony injuries such as healed tibial stress fractures, in adults [31]. We previously found that injured adults with a variety of bony and soft tissue injuries combined have 6% lower vertical displacement of COM overall and less ankle sagittal excursion during the gait cycle than healthy counterparts [37]. A novel finding from this study is that injured pediatric runners have higher cadence values but do not mitigate vGRF or VALR, narrow step widths or step lengths compared to healthy or adult counterparts (Table 2 and Table 3). In alignment with our data, studies on children and adolescents with painful calcaneal apophysitis demonstrate a higher step frequency compared to healthy counterparts [38]. In adolescents with bony injury, peak GRF and pelvic drop exist [17]. For rehabilitation purposes, pediatric runners may be able to adjust some gait features independently, like cadence, but require specific training in modifying impact load rates (using ‘soft’ landings), shortening step lengths, and widening stride widths [39]. These rehabilitation targets may improve vertical alignment of lower extremity joints, control pelvic motion, and better manage cumulative mechanical stresses on bones and tendons. For adults, focusing on pre-activation of lower extremity muscles (e.g., hip flexors, hamstrings, gluteal muscles, gastrocnemius) during the swing phase may enhance energy absorption after impact and help soften VALR. Additionally, an emphasis on a faster cadence may reduce step length, peak hip adduction, peak knee extensor moment, braking impulse, peak hip flexion, and patellofemoral joint stress [40], as well as frontal plane knee angle variability [41]. These findings can be directly implemented into clinical settings as part of tailored therapeutic programs for running athletes.

Limitations and Future Directions. Several study limitations deserve comment. First, this study is cross-sectional and cannot provide causal effects of biomechanics on recent injury. Second, there was likely variation in the stages of neuromotor maturation among runners in the pediatric groups, which may have contributed to variability in specific parameters. Future prospective research in larger groups of young runners across the developmental spectrum would provide insight into the gait strategies used as neuromotor pathways mature. Third, it is unclear how the recent history of different types of injuries affects biomechanics by age. To address this issue, larger samples of runners with either specific soft tissue injuries (e.g., tendinopathies, piriformis syndrome, IT band syndrome, calf pain) and bony injuries (e.g., metatarsal, tibial, fibular stress fractures) would greatly advance our understanding of adaptive responses to injury. Combined with electromyogram activity to capture muscle activation patterns in response to pain or injury, this information could contribute to considerable gains in knowledge of this area. Fourth, how pediatric and adult runners adjust biomechanics in response to fatigue is unknown at this time; systematic reviews show that decreased leg stiffness, increased knee flexion at initial contact, and increased maximum knee flexion occur in novice runners [42]. Future studies could advance the science by capturing real-time changes in these kinetic and kinematic parameters in both young and adult runners while they are in the outdoor training environment. Finally, our sample size for adult non-injured runners (*n* = 13) was relatively small, and there was a greater gender bias (Females = 76.9%), with the other three groups having a more equal distribution. Given these characteristics of our convenience sample, we are unable to rule out the effect of gender on certain biomechanical parameters between these groups. Future efforts should aim for equal gender distribution to rule out sex-specific differences.

## 5. Conclusions

Pediatric and adult runners exhibit distinct gait strategies that are not significantly or differentially impacted by injury status. Pediatric runners with injury reduce gait cycle timing variance and control pelvic rotation, whereas injured adult runners had lower GRF and longer stance time, indicating force mitigation strategies while in stance. There may be a need for age-specific rehabilitation and gait retraining approaches in runners.

## Figures and Tables

**Table 1:** Baseline characteristics. Values are means ± SD or % of the group. *p* values represent the group-by-time interactions.

	Pediatric		Adult			
Variable	Non-Injured (*n* = 23)	Injured (*n* = 96)	Non-Injured (*n* = 13)	Injured (*n* = 75)	*p*	η^2^ or Φ
Female (%)	47.8	43.8	76.9	58.7	0.012	0.017
Age (yr)	14.7 ± 1.7	15.1 ± 1.3	30.2 ± 3.3	29.4 ± 3.5	<0.001	0.009
Height (cm)	167.9 ± 8.9	167.7 ± 9.9	168.7 ± 8.5	171.1 ± 9.2	0.455	0.003
Weight (kg)	57.3 ± 11.8	56.8 ± 10.4	66.0 ± 10.3	69.1 ± 16.3	0.464	0.003
BMI (kg/m^2^)	20.2 ± 3.4	20.1 ± 2.6	23.2 ± 2.6	23.2 ± 3.3	0.933	0.001
Running history						
Weekly runs (#)	5.4 ± 3.7	4.6 ± 1.8	4.3 ± 1.7	3.7 ± 2.1	0.831	0.001
Volume (km/wk)	32.6 ± 21.4	34.6 ± 24.0	47.3 ± 34.1	32.8 ± 29.0	0.129	0.012
Speedwork (% yes)	47.8	55.9	30.0	50.7	0.469	0.394
Competing (% yes)	60.9	68.7	61.5	46.7	0.023	0.023
Shoe wear						
Heel height (cm)	33.1 ± 4.1	32.5 ± 7.1	31.4 ± 5.7	30.1 ± 8.1	0.864	0.001
Heel-to-toe drop (mm)	9.3 ± 2.1	8.2 ± 3.3	5.6 ± 3.1	7.7 ± 3.5	0.042	0.023
Weight (oz)	9.2 ± 1.5	9.0 ± 1.3	8.3 ± 1.4	8.8 ± 1.5	0.239	0.008
Cross training practices (%)						
Strength training						
Lower body	34.8	55.2	76.9	70.7	0.004	0.003
Upper body	34.8	60.4	76.9	72.0	0.013	0.011
Swimming	8.7	34.4	15.4	33.3	0.879	0.844
Cycling	13.0	46.9	38.5	54.7	0.093	0.088
Yoga/Pilates	13.0	10.4	23.1	25.3	0.009	0.008
Foot strike (%)						
Rearfoot	87.0	78.1	46.2	68.0		
Midfoot	13.0	18.8	46.2	25.3		
Forefoot	0.0	3.1	7.7	6.7	0.042	0.042
Injury type (%)						
Bone	---	61.5	---	47.3	0.050	0.046
Soft tissue	---	48.1	---	68.0	0.025	0.020

BMI = body mass index.

**Table 2:** Temporal spatial parameters of gait. Values are means ± SD. *p* values represent the group X time interactions.

	Pediatric		Adult		*p*			η^2^
Variable	Non-Injured (*n* = 23)	Injured (*n* = 96)	Non-Injured (*n* = 13)	Injured (*n* = 75)	Injury	Age	Intxn	
Velocity (km/h)	11.1 ± 1.8	10.7 ± 2.2	9.9 ± 1.7	10.0 ± 1.7	0.602	0.012	0.503	0.002
Cadence (step/min)	168 ± 11	170 ± 12	173 ± 12	167 ± 9	0.518	0.386	0.044	0.020
COM vertical displacement (cm)	9.5 ± 1.3	9.6 ± 1.6	9.2 ± 1.1	9.8 ± 1.3	0.262	0.836	0.405	0.004
COM M-L displacement (cm)	2.4 ± 0.6	2.5 ± 0.7	2.8 ± 0.7	2.8 ± 0.9	0.450	0.176	0.953	0.001
Step length (m)	1.02 ± 0.11	0.99 ± 0.18	0.91 ± 0.12	0.93 ± 0.16	0.722	0.281	0.583	0.00
Stride length (m)	1.5 ± 0.2	1.5 ± 0.3	1.4 ± 0.2	1.4 ± 0.2	0.447	0.280	0.273	0.007
Stride width (cm)	7.6 ± 2.2	7.6 ± 2.9	9.4 ± 2.3	9.1 ± 4.5	0.730	0.042	0.884	0.001
Stance time (% GC)	42.9 ± 3.7	42.3 ± 4.5	44.3 ± 3.1	44.4 ± 3.7	0.753	0.026	0.688	0.001
Stance time variance (SD)	0.005 ± 0.002	0.004 ± 0.001	0.003 ± 0.001	0.004 ± 0.002	0.557	<0.001	0.002	0.056
Swing time, average (% GC)	57.1 ± 3.7	57.6 ± 4.7	55.5 ± 3.1	55.4 ± 4.1	0.853	0.022	0.761	0.001
Swing time variance (SD)	0.010 ± 0.003	0.008 ± 0.003	0.005 ± 0.001	0.007 ± 0.002	0.465	<0.001	0.045	0.030

COM = center of mass; M-L = medial lateral; GC = gait cycle; SD = standard deviation; intxn = injury by age interaction.

**Table 3:** Kinetic parameters of gait, leg stiffness values, and joint moments. Values are means ± SD.

	Pediatric		Adult		*p*			η^2^
Variable	Non-Injured (*n* = 23)	Injured (*n* = 96)	Non-Injured (*n* = 13)	Injured (*n* = 75)	Injury	Age	Intxn	
vertical GRF (BW)								
Left	2.4 ± 0.2	2.6 ± 0.3	2.7 ± 0.5	2.4 ± 0.3	0.279	0.754	0.037	0.021
Right	2.5 ± 0.3	2.5 ± 0.3	2.6 ± 0.4	2.4 ± 0.3	0.417	0.911	0.013	0.120
VALR (BW/s)								
Left	73.1 ± 28.5	73.4 ± 27.9	58.7 ± 29.1	66.3 ± 25.5	0.315	0.022	0.337	0.005
Right	70.7 ± 21.5	75.1 ± 25.7	58.3 ± 29.0	66.8 ± 24.6	0.128	0.021	0.525	0.002
K_vert_ (N/cm)	148 ± 28	154 ± 35	184 ± 46	168 ± 33	0.318	0.017	0.309	0.006

SD = standard deviation; BW = body weight; GRF = ground reaction force; VALR = vertical average loading rate (impact loading); K_vert_ = vertical stiffness; intxn = injury by age interaction.

**Table 4:** Joint excursions during an average gait cycle. Values are expressed in degrees and are shown as means ± SD.

	Pediatric		Adult		*p*			η^2^
Variable	Non-Injured (*n* = 23)	Injured (*n* = 96)	Non-Injured (*n* = 13)	Injured (*n* = 75)	Injury	Age	Intxn	
Sagittal								
Ankle	53.1 ± 4.7	48.6 ± 7.4	46.7 ± 6.1	48.1 ± 7.8	0.325	0.015	0.036	0.027
Knee	85.9 ± 11.1	91.2 ± 12.3	82.7 ± 9.5	86.2 ± 14.3	0.079	0.098	0.778	0.001
Hip	57.9 ± 6.6	58.2 ± 9.9	52.4 ± 6.8	55.1 ± 8.7	0.312	0.276	0.967	0.001
Pelvis	8.1 ± 1.9	8.4 ± 2.3	9.0 ± 0.1	8.7 ± 2.3	0.983	0.146	0.532	0.002
Frontal								
Ankle	17.2 ± 4.2	15.4 ± 4.6	14.2 ± 5.6	15.2 ±5.3	0.484	0.181	0.251	0.008
Knee	10.1 ± 3.2	10.6 ± 3.7	10.4 ± 3.3	11.2 ± 5.3	0.544	0.514	0.940	0.001
Hip	20.6 ± 4.0	19.3 ± 5.3	20.3 ± 5.4	21.7 ± 5.7	0.900	0.395	0.172	0.011
Pelvis	10.5 ± 2.6	11.5 ± 2.9	11.3 ± 2.6	12.5 ± 3.4	0.044	0.180	0.655	0.001
Transverse								
Ankle	12.5 ± 3.2	13.0 ± 3.1	11.7 ± 3.5	11.2 ±3.2	0.960	0.037	0.477	0.003
Knee	16.8 ± 3.6	19.9 ± 5.4	20.0 ± 5.4	18.1 ± 2.4	0.109	0.014	0.111	0.016
Hip	17.0 ± 6.2	17.0 ± 5.0	16.0 ± 5.5	18.2 ± 6.9	0.943	0.127	0.124	0.014
Pelvis	17.3 ± 4.4	14.4 ± 5.3	12.0 ± 4.3	13.8 ± 4.8	0.472	0.005	0.036	0.027

intxn = injury by age interaction.

## Data Availability

Data sharing at this institution requires formal permission and Data Use Agreements. The raw data supporting the conclusions of this article could be made available by the authors upon reasonable request and review by the study team. This sharing is also contingent upon agreement with the University of Florida and appropriate data use agreements.

## Abbreviations

The following abbreviations are used in this manuscript:

GC	gait cycle
GRF	ground reaction force
VALR	vertical average load rate
LR	load rate
COM	center of mass
K_vert_	vertical leg stiffness
M-L	medial lateral
BMI	body mass index
